# Food Insecurity and Geriatric Hospitalization

**DOI:** 10.3390/ijerph16132294

**Published:** 2019-06-28

**Authors:** Rachel S. Bergmans, Briana Mezuk, Kara Zivin

**Affiliations:** 1 Department of Psychiatry, Medical School, University of Michigan, Ann Arbor, MI 48109, USA; 2 Department of Epidemiology, School of Public Health, University of Michigan, Ann Arbor, MI 48109, USA; 3 Department of Veterans Affairs, Health Services Research and Development, Center for Clinical Management Research, Ann Arbor, MI 48109, USA

**Keywords:** nutrition and aging, social gerontology, preventive geriatrics, internal medicine, psychological gerontology

## Abstract

Food insecurity (FI) has been associated with hospitalization, although the pathways underlying this relationship are poorly understood, in part due to the potential for a bidirectional relationship. This study aimed to determine associations of FI with concurrent and future hospitalization among older adults; mediation by depression and; whether hospitalization increased risk of FI. Participants came from the 2012 and 2014 waves of the Health and Retirement Study (HRS; *n* = 13,664). HRS is a prospective cohort representative of U.S. adults over the age of 50. Primary analyses included those who were not hospitalized in 2012 (*n* = 11,776). Not having enough money to buy necessary food or eating less than desired defined food insecurity. The Composite International Diagnostic Interview Short Form provided depression symptomology. Logistic and linear regression examined concurrent and longitudinal associations of FI in 2012 and 2014 with hospitalization in 2014. Path analysis tested mediation of FI with hospitalization frequency by depression symptomology. Finally, logistic regression examined whether hospitalization in 2012 was longitudinally associated with FI in 2014. FI was not associated with future hospitalization (odds ratio (OR) = 1.1; 95% confidence interval (CI) = 0.9–1.4), however; FI was associated with concurrent hospitalization status (OR = 1.4; 95% CI = 1.1–1.8). Depression symptomology explained 17.4% (95% CI = 2.8–32.0%) the association of FI with concurrent hospitalization frequency. Additionally, hospitalization was associated with becoming food insecure (OR = 1.5; 95% CI = 1.2–2.0). Findings may inform best practices for hospital discharge among older adults.

## 1. Introduction

Hospitalization, particularly among older adults, can be a pivotal event that signifies a decline in functional capacity, leading to intensification of healthcare intervention and lower quality of life [1]. Hospitalization is costly [2], and a potent determinant of transitions to more severe levels of disability among older adults [3]. This is of particular concern given that any disability or worsening disability among older adults increases the risk of mortality [4]. Although pre-existing conditions and multimorbidity contribute to inpatient hospital use [5], non-medical factors are also associated with healthcare utilization, including hospitalizations [6]. Given the negative consequences of hospitalization among older adults, one intervention approach has been improving elder care during hospitalization [7]. However, an alternative approach could be addressing upstream socioeconomic factors, like food insecurity. Food insecurity, which refers to limited or uncertain access to food of sufficient quantity or quality [8], has emerged as a social determinant of health that could be targeted to curb healthcare costs [9] and improve treatment outcomes [10] among older adults.

Risk of hospitalization due to food insecurity, and its converse—risk of food insecurity due to hospitalization—are not well established among older adults. Poor diet quality [11], depression [12], poor medication adherence [13] and poor diabetes management [14] are all considered negative health consequences of food insecurity that have also been associated with hospitalization among older adults [15,16,17,18]. Some evidence indicates that food insecurity is associated with hospitalization [19], while other research does not [20]. The dependence on cross-sectional data is a limitation of prior work; food insecurity is transient in nature and may have a bidirectional relationship with hospitalization. This study aimed to determine bidirectional associations of food insecurity with geriatric hospitalization. Additionally, to elucidate underlying pathways, this study examined mediation of the association between food insecurity and hospitalization by depression symptomology.

## 2. Materials and Methods

### 2.1. Sample

This study used data from the Health and Retirement Study (HRS) [21], an ongoing longitudinal cohort of older adults (aged 50 years or older) in the United States. The HRS Core survey is administered biennially and collects detailed information on health, financial assets, demographics and psychosocial factors. Prior to collecting data, HRS obtains written informed consent from respondents. HRS conducts interviews both in person and via telephone. The University of Michigan Institutional Review Board approved the HRS protocol (HUM00061128) and considered this study exempt from human subjects research since it is limited to secondary analyses of de-identified data.

**Figure 1:**
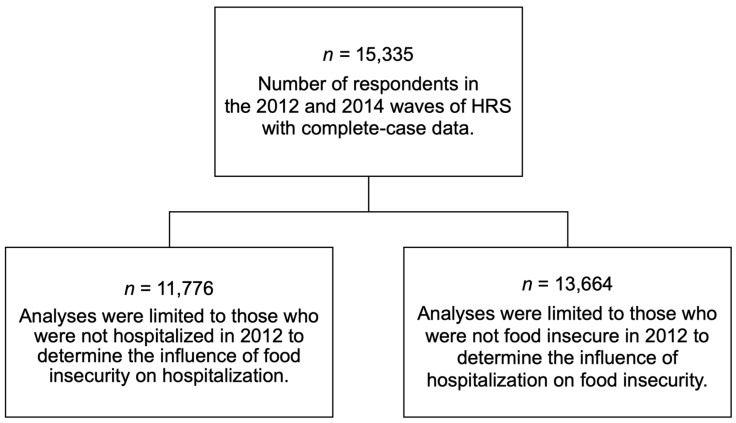
Flow chart for analytical samples within the 2012 and 2014 waves of the Health and Retirement Study (HRS).

This study included those who participated in both the 2012 and 2014 HRS waves (*n* = 16,502). Analyses were limited to those without missing data for variables of interest (*n* = 15,335; 93% of eligible individuals). Given that poor health status may increase vulnerability to food insecurity [22], analyses testing whether food insecurity was associated with future hospitalization included respondents who did not report hospitalization in 2012 (*n* = 11,776) to limit confounding due to baseline health care utilization. Analyses testing whether hospitalization was associated with future food insecurity included respondents who did not report food insecurity in 2012 (*n* = 13,664). Figure 1 provides a flow chart for this study’s analytical samples.

### 2.2. Measures

#### 2.2.1. Food Insecurity

The United States Department of Agriculture (USDA) 18-item food security tool is the gold standard for assessing food insecurity in the U.S. [23]. The HRS measured food insecurity in 2012 and 2014 using two questions adapted from a 2-item screener, which is considered a valid measure of food insecurity when compared to the USDA 18-item Food Security Tool [24]. First, participants were asked, “*In the last two years* [or, *since your last interview*]*, have you always had enough money to buy the food you need*”. Those that did not say “*yes*” were then asked, “*In the last 12 months, did you ever eat less than you felt you should because there wasn’t enough money to buy food?*” This study defined food insecurity as not having enough money to buy necessary food or eating less than desired. Food security referred to those who reported always having enough money to eat were food secure (reference group). This binary measure of food insecurity has been used in prior work testing associations of food insecurity with diabetic morbidity and depression symptomology [12]. This study treated food insecurity in 2012 as “Prior Food Insecurity” and food insecurity in 2014 as “Future Food Insecurity”.

#### 2.2.2. Hospitalization

HRS includes multiple questions to ascertain hospitalization status. The first question asked, “*In the last two years, have you been a patient in a hospital overnight?*” with options of yes or no. This item was used to determine recent hospitalization status, treated as a binary variable (yes vs. no = reference group). Among those who reported being hospitalized, HRS also asked, “*How many different times were you a patient in a hospital overnight in the last two years?*” and if the respondent specifically asked for further clarification, “*Include mental hospitals and sanitariums*”. This single item quantified the total number of hospitalizations, among those who were hospitalized, which ranged from 0 (indicating those who were not recently hospitalized) to 50. To improve interpretation of study findings, analyses treated hospitalization frequency as a standardized continuous variable (mean = 0, standard deviation = 1). This study treated hospitalization measures in 2012 as “Prior Hospitalization” and hospitalization measure in 2014 as “Future Hospitalization”.

#### 2.2.3. Depression

This study assessed depression both as a continuous measure of depression symptomology and as a binary indicator of current depression status. The HRS measures depression over the previous 12 months using the World Health Organization Composite International Diagnostic Interview Short Form (CIDI-SF). The CIDI-SF is based on the Diagnostic and Statistical Manual of Mental Disorders criteria for Major Depression [25], which requires that respondents report either symptoms of anhedonia or depressed mood for most of the day for most of a two-week period or more in order to be considered depressed. Those who endorse either of these screening items complete an additional seven symptoms: lost interest, feeling tired, change in weight, trouble with sleep, trouble concentrating, feeling down and thoughts of death. Depression symptom scores ranged from 0 to 7, and those who score ≥3 are considered to have experienced a major depressive episode [26]. Analyses of this study used the 2014 CIDI-SF.

#### 2.2.4. Covariates

Analyses accounted for several covariates to address potential confounding by demographic and socioeconomic factors. Demographic covariates included gender (female, male [reference group]), age group (51 to <65 [reference group], 65 to <75, ≥75), marital status (single or never married, widowed, separated or divorced, married [reference group]) and race/ethnicity (non-Hispanic White [reference group], non-Hispanic Black, other). Socioeconomic covariates included educational attainment during the first wave a respondent participated in HRS (<high school [reference group], high school degree or equivalent, some college, college degree and above), work status (work for pay vs. unemployed/retired/disabled/not in the labor force [reference group]) and household poverty-to-income ratio. Household poverty-to-income ratio is a continuous indicator of the ratio of an individual’s household income to the poverty level accounting for household size [27].

### 2.3. Statistical Approach

SAS version 9.4 (SAS Institute, Cary, NC, USA) [28] generated study sample descriptive statistics and model regression analyses. HRS study weights account for complex survey design, and the “domain” function in SAS to generate proper standard errors when limiting analyses to those who reported not being hospitalized or food insecure in 2012.

First, *X*^2^, *F* and Kolmogorov–Smirnov tests were used to generate descriptive statistics among those who did not report being hospitalized in 2012 by hospitalization status in 2014.

To test whether food insecurity was associated with future hospitalization, logistic regression modeled hospitalization status onto prior food insecurity (longitudinal association) and recent food insecurity (concurrent association); this analysis was done for all those who participated in HRS waves 2012 and 2014 (*n* = 15,335), as well as limited to those who did not report being hospitalized in 2012 (*n* = 11,776). Additionally, linear regression models assessed these associations for standardized hospitalization frequency.

Path analyses tested mediation of the association between food insecurity and hospitalization by depression symptomology in Stata 15 SE [29] among those who did not report being hospitalized in 2012. Path analysis is a type of structural equation modeling which tests both direct and indirect paths of associations for observed variables [30]. In order to meet assumptions of path estimation procedures, analyses treated depression symptomology as continuous, i.e., CIDI-SF scores ranging from 0 to 7.

In Stata, “svy: sem” paired with “nlcom” calculated the degree to which depression symptomology mediated the association of food insecurity with hospitalization frequency—and accounted for the complex survey design of HRS. Analyses consisted of two main paths: (1) food insecurity to depression symptomology score, and (2) depression symptomology score to hospitalization frequency. Covariates included in path analyses included gender, age group, marital status, race/ethnicity, educational attainment, household poverty-to-income ratio and work status.

To test the pathways from hospitalization to food insecurity, logistic regression modeled the odds of food insecurity in 2014 due to hospitalization in 2012. This analysis was done for all those who participated in HRS waves 2012 and 2014 (*n* = 15,335), as well as being limited to those who did not report food insecurity in 2012 (*n* = 13,664). Adjusted models accounted for gender, age group, marital status, race/ethnicity, educational attainment, household poverty-to-income ratio and work status.

## 3. Results

Table 1 provides descriptive statistics by 2014 hospitalization status among those not hospitalized in 2012 (*n* = 11,776). In 2014, 1988 respondents (17%) reported hospitalization; 1167 (9%) reported food insecurity in 2012; 1084 (8%) reported food insecurity in 2014 and; 873 (8%) met criteria for major depression in 2014.

Those who reported hospitalization in 2014 were more likely to be 75 years or older (25% vs. 13% of those not hospitalized) or widowed (16% vs. 10% of those not hospitalized). Those hospitalized in 2014 were also more likely to have lower educational attainment, not work for pay, and a lower household income-to-poverty ratio than those who were not hospitalized in 2014. Food insecurity status in 2012 did not differ by hospitalization status in 2014. In contrast, 10% of those hospitalized in 2014 also reported food insecurity over this same time period; whereas only 7% of those not hospitalized in 2014 reported food insecurity. Lastly, those who were hospitalized were more likely to meet criteria for major depression (13% vs. 7% of those not hospitalized).

Table 2 shows associations of food insecurity with hospitalization status. Before excluding those who reported a hospitalization in 2012, food insecurity was associated with future hospitalization status (odds ratio (OR) = 1.4; 95% confidence interval (CI) = 1.2–1.7). When excluding those who were previously hospitalized in 2012, food insecurity was no longer associated with future hospitalization status (OR = 1.1; 95% CI = 0.9–1.4). However, there was a concurrent association between food insecurity and hospitalization both before excluding those who were previously hospitalized in 2012 (OR = 1.7; 95% CI = 1.4–2.0) and after (OR = 1.4; 95% CI = 1.1–1.8).

Table 3 presents associations of food insecurity with hospitalization frequency. Before excluding those who were hospitalized in 2012, food insecurity was associated with future hospitalization frequency (*β* = 0.16; 95% CI = 0.08–0.24). After excluding those who were previously hospitalized in 2012, food insecurity was no longer associated with future hospitalization frequency (*β* = 0.02; 95% CI = −0.03–0.08). The association of food insecurity with concurrent hospitalization frequency was present before excluding those previously hospitalized in 2012 (*β* = 0.28; 95% CI = 0.17–0.38) and after (*β* = 0.11; 95% CI = 0.03–0.19).

**Figure 2:**
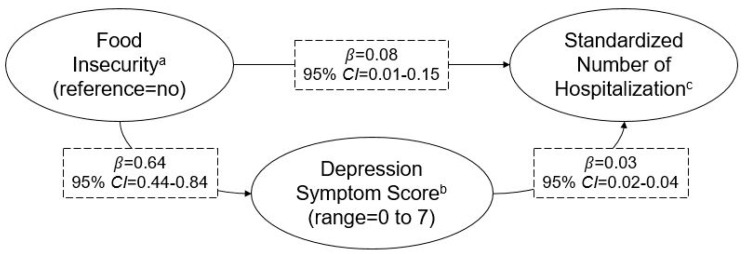
Mediation of the concurrent association between food insecurity and hospitalization frequency by depression symptomology among older adults. Depression symptomology explained 17.4% (95% confidence interval (CI) = 2.8–32.0%) of the concurrent association between food insecurity and hospitalization frequency. Data come from waves 2012 and 2014 of the Health and Retirement Study and limited to those who were not hospitalized in 2012. *β*’s represent direct effects and adjust for gender, age group, race/ethnicity, marital status, educational attainment, work status and household income-to-poverty ratio. ^a^ Collected in 2014 and assessed over the prior 24 months; those who reported that they could not always afford food due to lack of financial resources or ate less than desired were considered food insecure. ^b^ Collected in 2014 and assessed over the prior 12 months using the World Health Organization Composite International Diagnostic Interview Short Form (CIDI-SF). ^c^ Collected in 2014, assessed over the prior 24 months and standardized (mean = 0, standard deviation = 1).

Given that food insecurity was not associated with hospitalization longitudinally (Table 2 and Table 3), this study only tested mediation by depression symptomology for the concurrent association between food insecurity and hospitalization frequency—presented in Figure 2. Similar to findings from linear regression, recent food insecurity was associated with recent hospitalization frequency (*β* = 0.08; 95% CI = 0.01–0.15). Additionally, recent food insecurity was associated with depression symptomology (*β* = 0.64; 95% CI = 0.44–0.84). Depression symptomology was also associated with recent hospitalization frequency (*β* = 0.03; 95% CI = 0.02–0.04). Depression symptomology explained 17.4% (95% CI = 2.8–32.0%) of the concurrent relationship between food insecurity and hospitalization frequency.

Table 4. presents associations of hospitalization with food insecurity. Prior to excluding those who reported food insecurity in 2012, there were higher odds of food insecurity in 2014 due to hospitalization in 2012 (OR = 1.7; 95% CI = 1.3–2.1). This association remained when excluding those who reported food insecurity in 2012 (OR = 1.5; 95% CI = 1.2–2.0).

## 4. Discussion

In a nationally representative sample of older U.S. adults, food insecurity was not associated with future hospitalization. Food insecurity was associated with concurrent hospitalization status and concurrent hospitalization frequency. Depression partially mediated the relationship of food insecurity with concurrent hospitalization frequency. Additionally, hospitalization was associated with becoming food insecure.

Results demonstrated that food insecurity was not associated with future hospitalization. Similarly, in a sub-sample of those aged over 65 years old living in the U.S. southern state of Georgia, food insecurity was not associated with inpatient hospital stays [19]. When food insecurity is measured at a single time-point, as in this study, transient food insecurity cannot be separated from cases of chronic food insecurity. Perhaps only persistent and sustained periods of food insecurity lead to the coping and biological changes that increase risk of hospitalization.

In this study, food insecurity was associated with concurrent hospitalization. It is possible that food insecurity may only be associated with a risk of hospitalization in the short term. This is consistent with prior work, which demonstrated that the depletion of financial resources is associated with monthly peaks in hypoglycemia hospitalizations among those with diabetes [15].

Findings of this study also indicated that the concurrent relationship of food insecurity and hospitalization was mediated by depression symptomology. Stigma and poor diet quality, associated with food insecurity, can contribute to depression [31,32]. Depression may reduce self-care and management of medical comorbidities, thus resulting in more frequent hospitalization [33,34]. Alternatively, hospitalization may lead to the onset of depressive symptoms, which could reduce one’s capacity to manage financial and food resources. Further work is needed to disentangle the relationship of food insecurity with hospitalization, and the mediating role of depression.

In this study, hospitalization was associated with becoming food insecure. Hospitalization is costly [2], which could strain financial savings or resources, and is associated with greater functional impairment among older adults [3]—both of which are related to food insecurity [8]. This finding has important implications given that food insecurity is a social determinant of health with a host of negative mental and physical health consequences [11,12,13,14]. Prior work has consistently demonstrated that food assistance programs (e.g., vouchers, home-delivered meals) can reduce food insecurity and improve health [35,36,37], including among home-bound older adults [38]. Identifying older adults at risk of food insecurity during hospitalization discharge and enrolling them in food assistance programs may yield health benefits.

### Limitations

Cross-sectional analyses of this study prevent drawing causal conclusions regarding concurrent associations of food insecurity and hospitalization. In 2014, food insecurity and hospitalizations were assessed over the previous 24 months, and depression symptomology was assessed over the previous 12 months; thus, mediation by depression symptomology may be underestimated. Additionally, those who were hospitalized may have had a harder time recalling food insecurity or depression symptomology, which would result in underestimating associations.

## 5. Conclusions

Findings indicated that food insecurity was not associated with future hospitalization. However, food insecurity was associated with concurrent hospitalization, and this relationship was partially mediated by depression symptomology. Additionally, when examining the potential bidirectional relationship of food insecurity with hospitalization, hospitalization was associated with future food insecurity.

## Figures and Tables

**Table 1:** Descriptive statistics by 2014 hospitalization status ^a,b^.

Characteristics	Total Sample	Hospitalized		*p* Value ^c^
		No	Yes	
	*n* = 11,776	*n* = 9788	*n* = 1988	
Female	6858 (54.7)	5715 (54.8)	1143 (54.3)	0.7075
Age Category				<0.0001
51 to <65	6036 (58.9)	5300 (61.7)	736 (43.5)	
65 to <75	3188 (25.9)	2578 (24.8)	610 (31.6)	
75 to 106	2552 (15.2)	1910 (13.4)	642 (25.0)	
Race/Ethnicity				0.2995
Non-Hispanic White	7519 (78.0)	6169 (77.8)	1350 (79.1)	
Non-Hispanic Black	2222 (9.5)	1869 (9.5)	353 (9.7)	
Other	2035 (12.5)	1750 (12.7)	285 (11.2)	
Marital Status				<0.0001
Married	6998 (62.4)	5898 (63.6)	1100 (55.8)	
Separated or divorced	1724 (14.6)	1439 (14.4)	285 (15.5)	
Widowed	1800 (11.3)	1411 (10.4)	389 (16.1)	
Single or never married	1254 (11.7)	1040 (11.5)	214 (12.6)	
Baseline educational attainment ^d^				<0.0001
<High school	1979 (11.7)	1618 (11.3)	361 (13.5)	
High school degree or equivalent	3894 (31.3)	3158 (30.0)	736 (37.9)	
Some college	2962 (25.9)	2476 (26.1)	486 (24.8)	
College degree and above	2941 (31.1)	2536 (32.5)	405 (23.7)	
Work for Pay	5342 (52.3)	4667 (54.5)	675 (40.6)	<0.0001
Income-to-Poverty Ratio, median, (minimum, maximum) ^e^	3.8 (0.0, 340.3)	4.0 (0.0, 340.3)	3.2 (0.0, 333.3)	<0.0001
Food Security Status ^f^				
Previously food insecure	1167 (8.5)	960 (8.4)	207 (9.2)	0.4054
Recently food insecure	1084 (7.6)	874 (7.2)	210 (9.6)	0.0020
Depression ^e,g^				
Symptom score, median, (minimum, maximum)	0 (0, 7)	0 (0, 7)	0 (0, 7)	<0.0001
Met criteria for major depression	873 (7.6)	630 (6.7)	243 (12.7)	<0.0001

^a^ Data came from respondents who participated in waves 2012 and 2014 of the Health and Retirement Study and did not report being hospitalized in 2012. ^b^ All variables measured in 2012 wave and table values represent weighted column percentages unless otherwise indicated. ^c^ *x*^2^ or *F* test. ^d^ Measured the first year a respondent participated in HRS. ^e^ Values were not normally distributed based on the Kolmogorov–Smirnov test (*p*-value < 0.01). ^f^ Assessed over the past 24 months; those who reported that they could not always afford food due to lack of financial resources or ate less than desired were considered food insecure. Previous food insecurity measured in 2012, recent food insecurity measured in 2014. ^g^ Assessed over the past 12 months using the Composite International Diagnostic Interview Short Form (CIDI-SF), those with a score >3 were categorized as having experienced a major depressive episode.

**Table 2:** Odds ratios and 95% confidence intervals for the association between food insecurity and hospitalization status ^a,b^.

**Complete-Case 2012 and 2014 Sample (*n* = 15,335)**						
**Model**	**2012 Food Insecurity ^c^**		***p*-Value ^d^**	**2014 Food Insecurity ^c^**		***p*-Value ^d^**
	**No**	**Yes**		**No**	**Yes**	
Crude	Reference	1.4 (1.2, 1.7)	<0.0001	Ref.	1.7 (1.4, 2.0)	<0.0001
Adjusted ^e^	Reference	1.4 (1.2, 1.7)	0.0005	Ref.	1.7 (1.4, 2.0)	<0.0001
**Restricted to Those Not Hospitalized in 2012 (*n* = 11,776)**						
**Model**	**2012 Food Insecurity ^c^**		***p*-Value ^d^**	**2014 Food Insecurity ^c^**		***p*-Value ^d^**
	**No**	**Yes**		**No**	**Yes**	
Crude	Reference.	1.1 (0.9, 1.4)	0.41	Ref.	1.4 (1.1, 1.7)	0.0020
Adjusted ^e^	Reference.	1.1 (0.9, 1.4)	0.39	Ref.	1.4 (1.1, 1.8)	0.0017

^a^ Data came from respondents who participated in waves 2012 and 2014 of the Health and Retirement Study. ^b^ Odds of reporting being hospitalized (yes, no (reference group)) over the past 24 months, assessed in 2014. ^c^ Assessed over the past 24 months; those who reported that they could not always afford food due to lack of financial resources or ate less than desired were considered food insecure. ^d^ Type III. ^e^ Model accounts for gender, age group, race/ethnicity, marital status, educational attainment, work status and household income-to-poverty ratio.

**Table 3:** *β*’s and 95% confidence intervals for the association between food insecurity and standardized hospitalizations frequency ^a,b^.

**Complete-Case 2012 and 2014 Sample (*n* = 16,455)**						
**Model**	**2012 Food Insecurity ^c^**		***p*-Value**	**2014 Food Insecurity ^c^**		***p*-Value**
	**No**	**Yes**		**No**	**Yes**	
Crude	Reference	0.19 (0.12, 0.27)	<0.0001	Ref.	0.31 (0.20, 0.41)	<0.0001
Adjusted ^d^	Reference	0.16 (0.08, 0.24)	0.0001	Ref.	0.28 (0.17, 0.38)	<0.0001
**Restricted to Those Not Hospitalized in 2012 (*n* = 11,776)**						
**Model**	**2012 Food Insecurity ^c^**		***p*-Value**	**2014 Food Insecurity ^c^**		***p*-Value**
	**No**	**Yes**		**No**	**Yes**	
Crude	Reference	0.03 (−0.02, 0.08)	0.24	Ref.	0.11 (0.04, 0.18)	0.0034
Adjusted ^d^	Reference	0.02 (−0.03, 0.08)	0.41	Ref.	0.11 (0.03, 0.19)	0.0065

^a^ Data came from respondents who participated in waves 2012 and 2014 of the Health and Retirement Study. ^b^ Number of times participant reported being hospitalized in 2014 over the past 24 months, and standardized (mean = 0, standard deviation = 1). ^c^ Assessed over the past 24 months; those who reported that they could not always afford food due to lack of financial resources or ate less than desired were considered food insecure. ^d^ Model accounts for gender, age group, race/ethnicity, marital status, educational attainment, work status and household income-to-poverty ratio.

**Table 4:** Odds ratios and 95% confidence intervals for the influence of hospitalization on food insecurity ^a,b^.

**Complete-Case 2012 and 2014 Sample (*n* = 15,335)**			
**Model**	**2012 Hospitalization ^c^**		***p*-Value ^d^**
	**No**	**Yes**	
Crude	Reference	1.7 (1.5, 2.1)	<0.0001
Adjusted ^e^	Reference	1.7 (1.3, 2.1)	<0.0001
**Restricted to Those Not Food Insecure in 2012 (*n* = 13,664)**			
**Model**	**2012 Hospitalization ^c^**		***p*-Value ^d^**
	**No**	**Yes**	
Crude	Reference	1.5 (1.2, 2.0)	0.0006
Adjusted ^e^	Reference	1.5 (1.2, 2.0)	0.0020

^a^ Data came from respondents who participated in waves 2012 and 2014 of the Health and Retirement Study. ^b^ Food insecurity assessed over the past 24 months in 2014; those who reported that they could not always afford food due to lack of financial resources or ate less than desired were considered food insecure. ^c^ Odds of reporting being hospitalized (yes, no (reference group)) over the past 24 months. ^d^ Type III. ^e^ Model accounts for gender, age group, race/ethnicity, marital status, educational attainment, work status and household income-to-poverty ratio.
